# Antiemetic treatment of hyperemesis gravidarum in 1,064 Norwegian women and the impact of European warning on metoclopramide: a retrospective cohort study 2002–2019

**DOI:** 10.1186/s12884-022-04777-x

**Published:** 2022-06-02

**Authors:** Hilde Erdal, Lone Holst, Kristine Heitmann, Jone Trovik

**Affiliations:** 1 Centre for Pharmacy, Department of Global Public Health and Primary Care, Faculty of Medicine, University of Bergen, Årstadveien 17, 5009 Bergen, Norway; 2 Regional Medicines Information and Pharmacovigilance Centre, Department of Clinical Biochemistry and Pharmacology, Haukeland University Hospital, Bergen, Norway; 3 Department of Gynecology and Obstetrics, Haukeland University Hospital, Bergen, Norway; 4 Department of Clinical Science, Faculty of Medicine, University of Bergen, Bergen, Norway

**Keywords:** Hyperemesis gravidarum, Antiemetics, Pregnancy, Termination of pregnancy, Metoclopramide, Ondansetron, Meclizine, Prochlorperazine, EMA, Guidelines

## Abstract

**Background:**

Women suffering from severe nausea and vomiting during pregnancy, hyperemesis gravidarum, have poor quality of life and increased risk of potentially fatal maternal and fetal complications. There is increasing and reassuring knowledge about safety of antiemetics in pregnancy. In 2013, the European Medical Agency (EMA) issued a warning on metoclopramide limiting treatment to maximum five days. Metoclopramide was the most used antiemetic in pregnancy at the time the warning was implemented in the Norwegian hyperemesis guidelines (2014). We aimed at describing changes in the treatment of hyperemesis over time, including changes associated with the EMA warning.

**Methods:**

Retrospective chart review of all women hospitalized for hyperemesis gravidarum with metabolic disturbances between 01/Jan/2002 and 31/Dec/2019 at a university hospital serving nearly 10% of the pregnant population in Norway. Time-series analysis described changes over time and interrupted time series analysis quantified changes in treatment and clinical outcomes related to the EMA warning.

**Results:**

In total, 1,064 women (1.2% of the birthing population) were included. The use of meclizine, prochlorperazine, and ondansetron increased during 2002–2019. This led to a yearly increase in the percentage of women using any antiemetic of 1.5% (95%CI 0.6; 2.4) pre-hospital, 0.6% (95%CI 0.2; 1.1) during hospitalization, and 2.6% (95%CI 1.3; 3.8) at discharge. Overall, only 50% of the women received antiemetics pre-hospital. Following the EMA warning, prehospital use of metoclopramide dropped by 30% (95%CI 25; 36), while use of any antiemetic pre-hospital dropped by 20% (95%CI 5.7; 34). In timely association, we observed a decrease in gestational age (-3.8 days, 98.75%CI 0.6; 7.1) at first admission, as well as indication of increased rate of termination of pregnancy with an absolute increase of 4.8% (98.75%CI 0.9; 8.7) in 2014.

**Conclusion:**

During 2002–2019, the overall use of antiemetics in treatment of hyperemesis increased. The EMA-warning on metoclopramide in 2013 temporarily limited pre-hospital antiemetic provision associated with hospitalization at lower gestational length and indication of an increase in termination of pregnancy.

## Background

Nausea and vomiting are common pregnancy complaints, affecting nearly 70% of pregnant women to some degree, from mild to severe [1]. Hyperemesis gravidarum (HG) is the most severe form of pregnancy sickness which inhibits normal fluid and/or food intake and strongly limits activities of daily living [2]. A global meta-analysis and a large UK population study found a prevalence of HG of 1.1% and 1.5%, respectively [1, 3].

Secondary to persisting nausea and vomiting, HG can lead to dehydration, electrolyte imbalance, weight loss, and metabolic disturbances, which can be life-threatening if left untreated [4]. In addition to maternal risk of anemia, hypertension, coagulopathy, and preeclampsia, HG is associated with low fetal birth weight and preterm birth [3, 4]. HG-sufferers report symptoms that can be nearly unbearable, illustrated by the findings that half of the HG-patients in a large UK survey considered to terminate the pregnancy, and one in four experienced occasional suicidal ideation [5]. Additionally, there is a considerable risk of recurrent HG in future pregnancies [6, 7] and many who have had HG are reluctant to become pregnant again [7, 8].

First-line antiemetics for nausea and vomiting during pregnancy (NVP)/HG have traditionally included antihistamines (meclizine, promethazine, and cyclizine), and dopamine antagonists (prochlorperazine, and chlorpromazine) as these have reassuring evidence of safety in pregnancy [9–12]. None of these has been demonstrated as significantly better in reducing NVP [13]. If moderate NVP-symptoms persist despite complementary treatment, guidelines in general recommend these first-line antiemetics to be offered [4] to reduce need for health care visits, hospital inpatient treatment, and to alleviate the disease burden for the affected women [14, 15]. For severe NVP or HG, ambulatory or inpatient hospital care are usually required to provide fluid and nutritional treatment [4]. The serotonin antagonist ondansetron is a second line antiemetic option due to suspicion of a small increased risk of orofacial clefts [9–12, 16, 17]. Prednisolone can be used as an antiemetic in refractory cases [9, 10]. Antiemetics are commonly prescribed off label, as many countries, including Norway, do not have NVP/HG as licensed indication [4].

In 2013, the European Medicines Agency (EMA) issued a warning regarding licensed use of the dopamine antagonist metoclopramide due to risk of extrapyramidal adverse effects limiting treatment duration to up to five days and reducing dose recommendations [18]. These recommendations were issued as a general precaution for metoclopramide by the Norwegian Medicines Agency in July 2013 [19]. Even though risk and benefit associated with use of metoclopramide for NVP/HG was not a part of the EMA safety review, the five-day limit was implemented in the Norwegian HG treatment guidelines in the 2014 update and UK green top guidelines in 2016 [9, 10]. Subsequently, despite a very good fetal safety profile, metoclopramide was considered a second-line option due to risk of maternal adverse effects. The dopamine antagonists prochlorperazine and chlorpromazine were preferred [9, 10, 20]. Between 2004 and 2017, 60% of all antiemetic prescription fills during pregnancy in the Norwegian Prescription Database (NorPD) were for metoclopramide [21], but changes in treatment provision for patients with HG related to the metoclopramide warning has not previously been investigated.

The aim of this study was to describe the antiemetic treatment provided for patients hospitalized for HG at a Norwegian university hospital and elucidate changes in patient characteristics and outcomes over time, including the impact of the EMA-warning on treatment and outcomes in patients with HG.

## Methods

### Setting & design

This retrospective cohort study was conducted by review of hospital patient files for all women admitted for treatment of HG at the Department of Obstetrics and Gynecology, Haukeland University Hospital, Bergen, Norway between 01/Jan/2002 and 31/Dec/2019. The Bergen Hospital Trust provides hospital service to approximately 10% of the Norwegian female population.

### Participants

Inclusion criteria were diagnosis of HG (International classification of disease version 10 (ICD-10) code O21.1 hyperemesis with metabolic complications) defined as excessive nausea and vomiting in pregnancy with at least two of the following three criteria: dehydration, weight loss (from pre-pregnancy weight), or electrolyte imbalance/ketonuria with first hospital admission prior to 20 weeks gestation. Exclusion criteria were other medical conditions causing nausea and vomiting, e.g. gastroenteritis. The inclusion- and exclusion criteria were assessed individually based on information in the patient’s hospital file and relevant data were cross-checked with the mandatory Norwegian pregnancy out-patient chart included in the hospital file.

### Measurements

Yearly HG admission rate at the hospital was calculated as number of included women by the date of first admission compared to the corresponding birthing population at Haukeland University Hospital registered in the Medical Birth Registry of Norway (MBRN) [22]. Gestational and maternal characteristics, relevant laboratory results, and treatment information were collected from the hospital records using a standardized data collection form. Body mass index (BMI, (kg)/height(m)^2^) was calculated from self-reported pre-pregnancy weight collected from the mandatory Norwegian antenatal card. Weight change at first admission was calculated as difference in percent of pre-pregnancy weight. History of HG was registered, and if not reported in her patient file, it was assumed that the woman did not have HG in previous pregnancies. For women with several HG-pregnancies during the study period, all pregnancies were included, but each pregnancy was considered independent in the analyses.

Gestational length on first admission and at birth was calculated based on due date estimated at the routine ultrasound examination in gestational week 17–20, which is the standard method in Norway [10]. If due date was missing (e.g. patients with miscarriage, or termination of pregnancy), gestational length was calculated from ultrasound examination performed on admission.

Use of all antiemetics documented in the patient file regarding use prior to, during, and after hospitalization was registered, including use of prednisolone for antiemetic purposes. Use of more than one antiemetic on the same day during hospital admission was registered as use of combination therapy with antiemetics.

### Statistical analyses

Changes in maternal characteristics and treatment over time were analyzed as time-series, i.e., chronologically sequenced data separated by equally spaced time points (year). Trends were assessed using ordinary least squares linear (OLS) regression with Newey-West standard errors with one lag (lag[1]), correcting for first order autocorrelation, and presented as yearly change with 95% confidence interval (CI). Use of combinations of antiemetics was presented as a time-series including combinations used by more than 50 women.

Interrupted time-series analysis (ITSA) was used to investigate the potential impact of the EMA warning from July 2013 [18] and the Norwegian HG treatment guidelines update in February 2014 [10] on: the proportion of women using metoclopramide, prochlorperazine, or any antiemetic prior to, during, and after hospitalization (all presented as % of admissions), and changes in clinical outcomes defined as: admission rate (% of births), rate of pregnancy termination (% of admissions), and yearly mean weight change (% of pre-pregnancy weight) and gestational age (days) at first admission. The ITSA-analyses were conducted specifying Newey-West lag[1] with intervention start point defined as January 1st 2014 on data modelled by year [23]. The ITSA-analyses generated coefficients for: pre-intervention trend as yearly change (β_1_); level change at the time of intervention (β_2_); change in trend post-intervention (β_3_); and post-intervention trend (β_1_ + β_3_). In addition to the p-value for the model, all estimates were presented with corresponding CI [23]. All tests were two-sided. Results on descriptive analyses regarding treatment changes were considered statistically significant if *p* < 0.05. For hypothesis tests on four clinically relevant outcome measures, the statistical significance limit was adjusted to *p* < 0.0125 according to the Bonferroni correction.

Data were analyzed using Stata, StataCorp. 2021. *Stata Statistical Software: Release 17*. College Station, TX: StataCorp LLC. This study is reported in adherence with the STROBE guidelines.

## Results

**Fig. 1:**
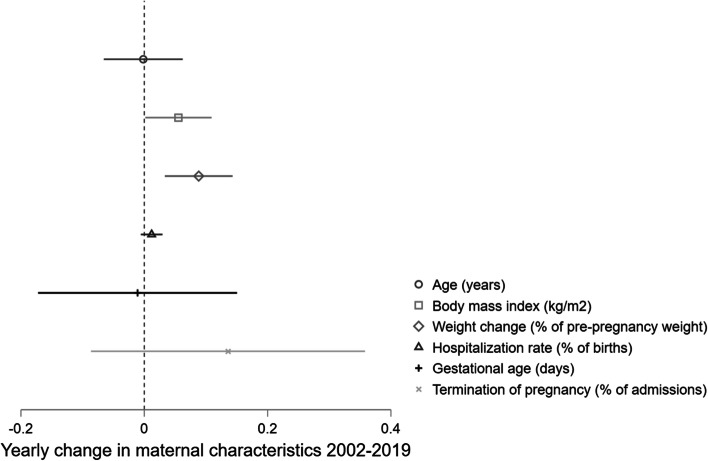
Changes in maternal characteristics in patients admitted for hyperemesis gravidarum at Haukeland University Hospital 2002–2019. Linear trends with Newey West standard errors accounting for one lag autocorrelation displayed as annual change (mark) and 95% confidence intervals (line). *% of pre-pregnancy weight

Between 2002 and 2019, 1,064 women were admitted for treatment of HG with metabolic disturbances at Haukeland University Hospital. With a total of 89,262 births, this corresponds to a yearly admission rate of 1.2%. Following the first hospitalization, 32% of the women were readmitted at least once. An overall increasing trend was observed in maternal pre-pregnancy BMI (95%CI 0.002; 0.11 kg/m^2^ yearly) while maternal weight change at first hospitalization decreased during the study period (95%CI 0.033; 0.14% of pre-pregnancy weight annually). Throughout the study period, termination of pregnancy was seen in a total of 61 women (5.7% of the study population). Further characteristics of the study population are described in Table 1, while changes over time are illustrated in Fig. 1.

**Table 1:** Characteristics of women admitted for treatment of hyperemesis gravidarum at Haukeland University Hospital 2002–2019, *n* = 1,064

	**Mean (SD)**
Age (years)	28.6 (5.2)
Pre-pregnancy BMI (kg/m^2^)^a^	24.5 (4.7)
Weight change at first admission (% of pre-pregnancy weight)	-5.8 (4.4)
Gestational length at first admission (days)^c^	66 (19)
	**Median (IQR)**
Gravidity^d^	2 (1; 3)
Parity^e^	1 (0; 1)
Previous pregnancies with hyperemesis^f^	1 (1; 2)
Number of hospitalizations	1 (1; 2)
Total number of days in hospital^g^	2 (1; 5)
Total number of antiemetics pre-hospital^h^	1 (1; 2)
Total number of antiemetics while hospitalized^i^	2 (1; 3)
	**n (%)**
Readmitted once or more	341 (32)
Termination of pregnancy	61 (5.7)

Abbreviations: *n* number, *SD* standard deviation, *IQR* interquartile range, *BMI* body mass index

^a^*n* = 19 missing values

^b^*n* = 16 missing values

^c^*n* = 2 missing values

^d^*n* = 4 missing values

^e^*n* = 1 missing value

^f^*n* = 320 (30%) had previous pregnancies with HG

^g^Number of days hospitalized, including readmissions

^h^*n* = 532 (50%) using antiemetics prior to first hospitalization for hyperemesis

^i^*n* = 1,016 (95%) using antiemetics during hospital stay, including readmissions

**Fig. 2:**
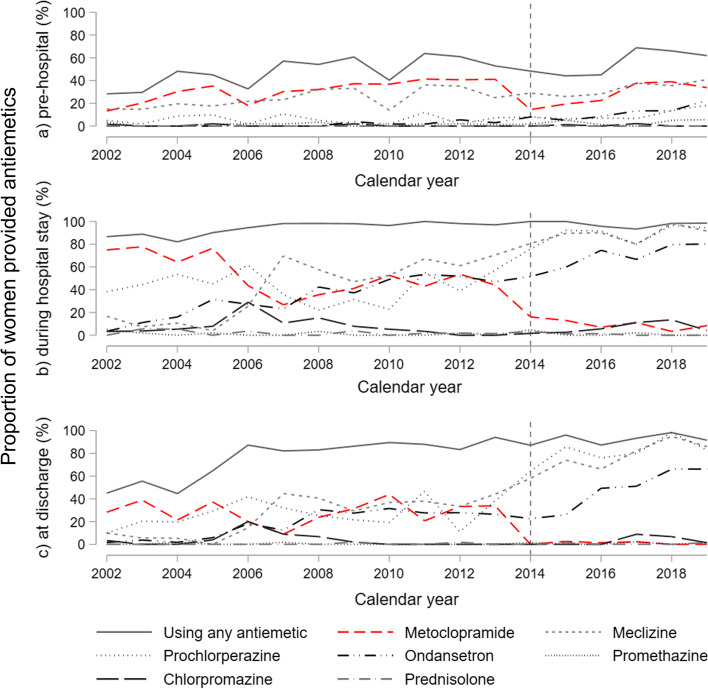
Use of antiemetics in patients admitted for hyperemesis gravidarum at Haukeland University Hospital 2002–2019. Time series from Haukeland University Hospital, *n* = 1,064

**Fig. 3:**
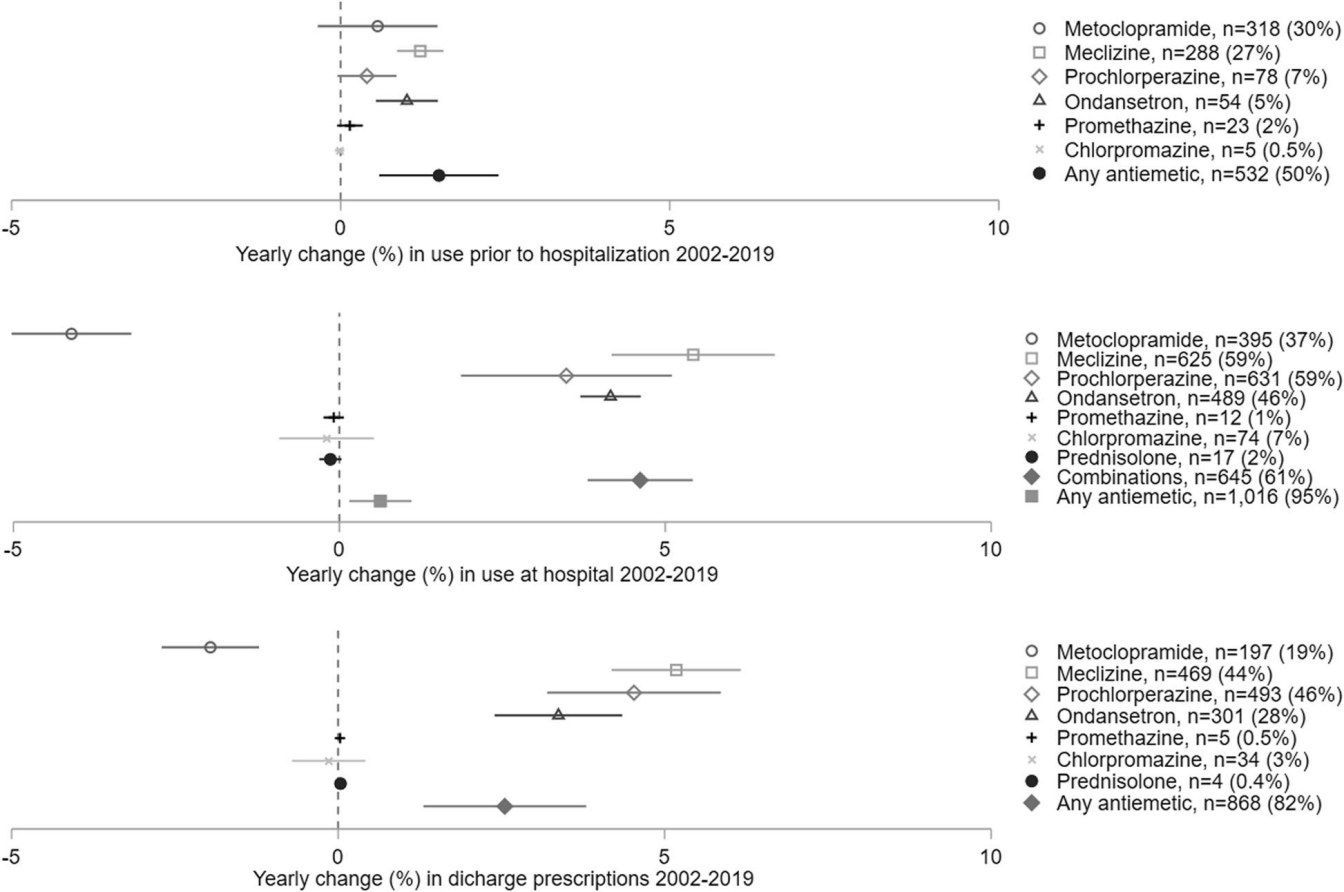
Changes in use of antiemetics in patients admitted for hyperemesis gravidarum at Haukeland University Hospital 2002–2019. Linear trends with Newey West standard errors accounting for one lag autocorrelation displayed as annual change (mark) in % with corresponding 95% confidence intervals (line). n(%) reflects the total number and percentage of patients using the antiemetic. Combination of antiemetics refers to use of more than one antiemetic on the same day during hospital admission

Use of antiemetics pre-, per, and post hospitalization increased during the study period, as illustrated in the time-series in Fig. 2 with corresponding trends displayed in Fig. 3. Overall, half of the women (50%) had used antiemetics prior to hospitalization, with increasing trend during the study period of 1.5% (95%CI 0.6; 2.4) yearly. Nearly all women (95%) were treated with antiemetics during their hospital stay. Similarly, prescriptions for antiemetics at discharge were registered in 81% of the women overall, with an increase of 2.6% (95%CI 1.3; 3.8) yearly. Use of meclizine, prochlorperazine, and ondansetron increased during the study period both pre-, per-, and post hospital (Figs. 2 and 3).

**Fig. 4:**
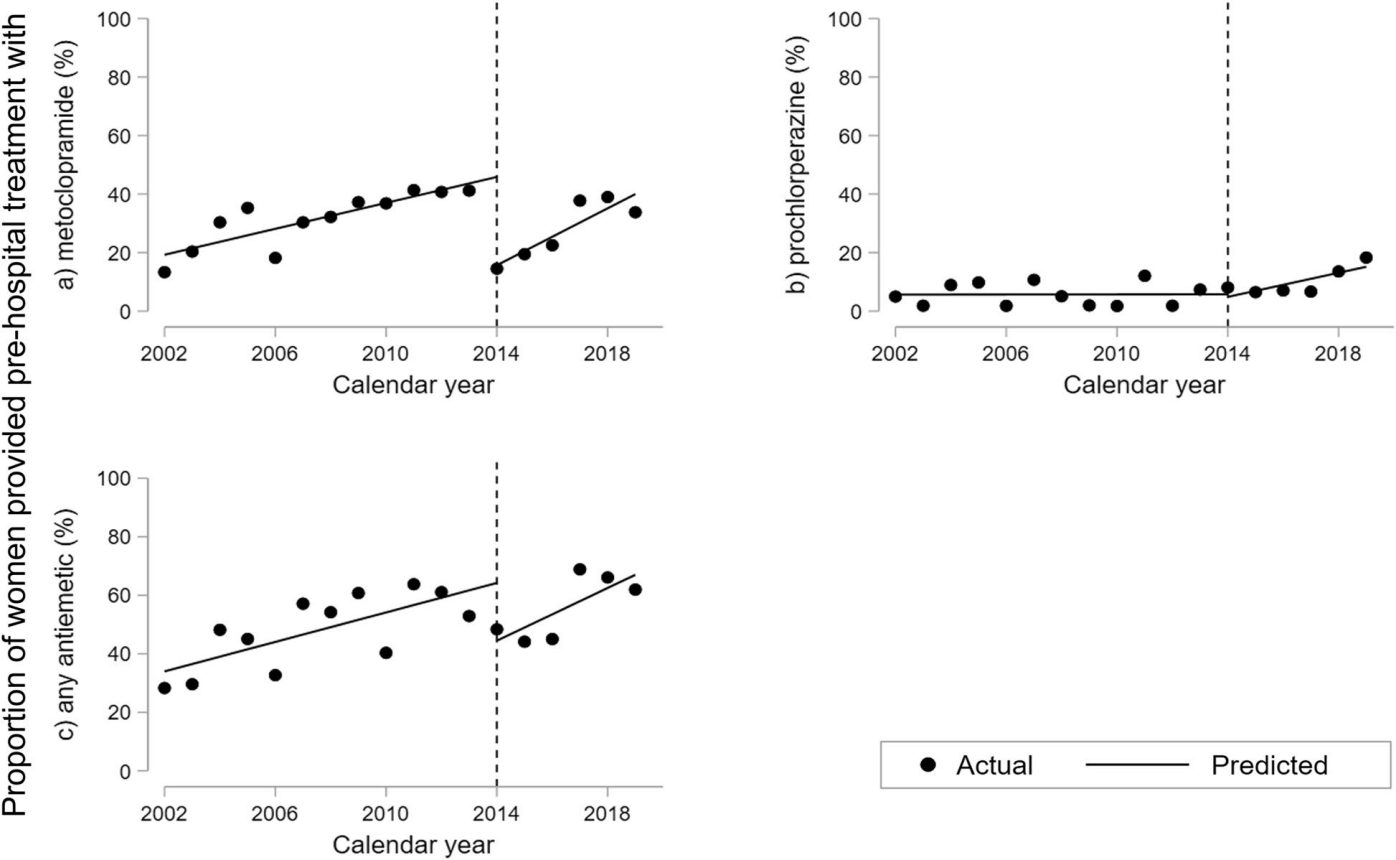
Interrupted time-series analysis of use of antiemetics prior to hospitalization for hyperemesis gravidarum 2002–2019. Percentage of 1.064 women hospitalized at Haukeland University Hospital using (**a**) metoclopramide, (**b**) prochlorperazine, and (**c**) any antiemetic prior to first hospitalization. Predicted line visualizes linear trend pre-intervention in 2014 with slope corresponding to yearly change (β_1_); vertical line illustrating level change in 2014 at the time of treatment guideline update (β_2_); predicted line after 2014 representing post-intervention trend (β_1_ + β_3_) applying Newey-West standard errors to adjust for first order autocorrelation

**Fig. 5:**
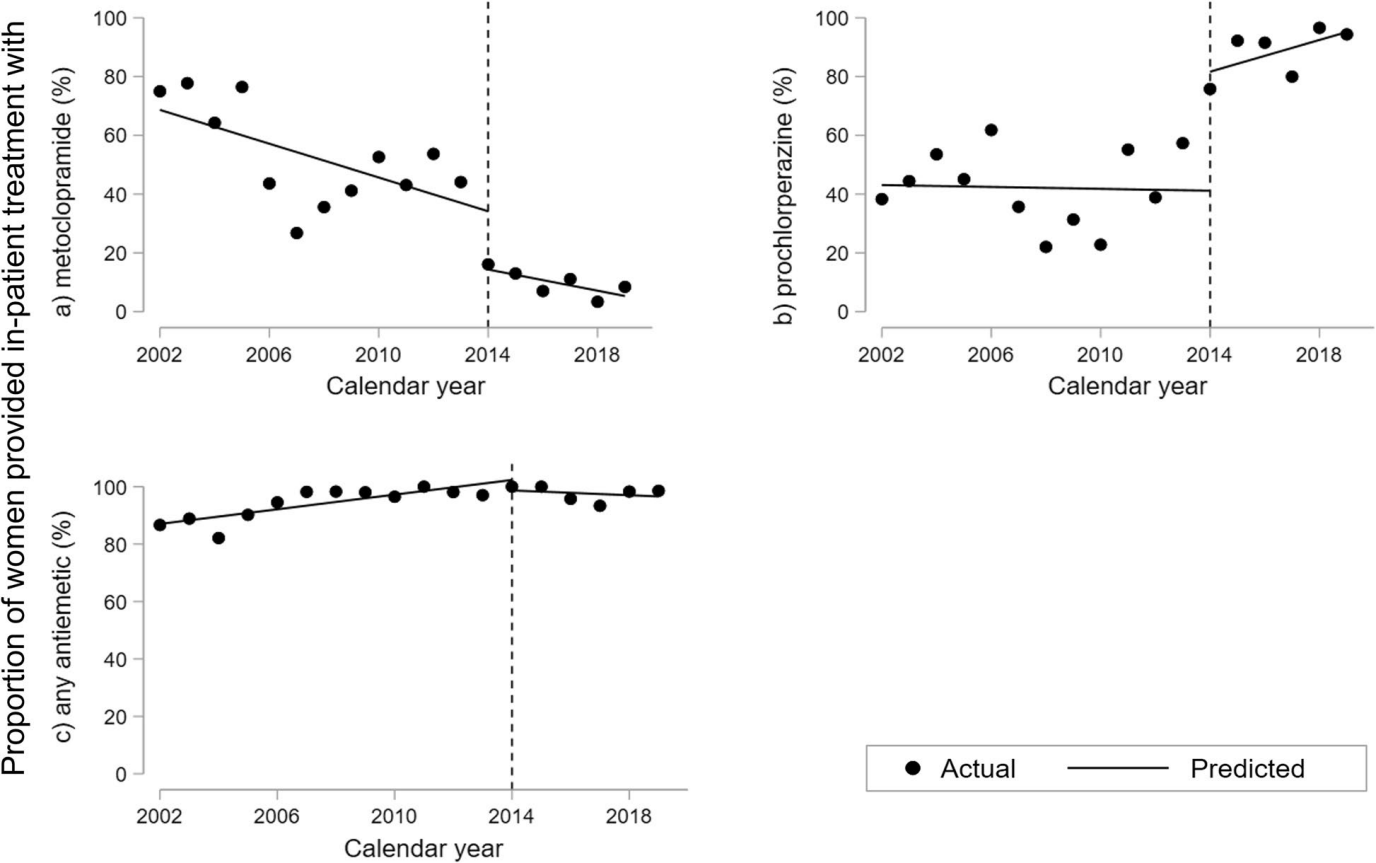
Interrupted time-series analysis of use of antiemetics while hospitalized for hyperemesis gravidarum 2002–2019. Percentage admitted to Haukeland University Hospital using (**a**) metoclopramide, (**b**) prochlorperazine, and (**c**) any antiemetic while hospitalized. Predicted line visualizes linear trend pre-intervention in 2014 with slope corresponding to yearly change (β_1_); vertical line illustrating level change in 2014 at the time of treatment guideline update (β_2_); predicted line after 2014 representing post-intervention trend (β_1_ + β_3_) applying Newey-West standard errors to adjust for first order autocorrelation

The results of the interrupted time-series analyses describing changes in use of metoclopramide, prochlorperazine and antiemetics overall are illustrated in Figs. 4 and 5 with estimates presented in Table 2. Despite a rising trend until 2013, we found an absolute reduction in the proportion receiving metoclopramide prior to hospitalization of 30.2% (95%CI 24.8; 35.6) in 2014, followed by a rise in the trend after the intervention adding up to a 4.86% (95%CI 2.68; 7.04) increase yearly from 2014 onwards (Fig. 4a, Table 2a). We found no change in pre-hospital use of prochlorperazine prior to or in 2014, but indication of a rising trend post-intervention of 2.06% (95%CI 0.48; 3.64) yearly (Fig. 4b, Table 2a). The proportion receiving any antiemetic prior to admission decreased by 19.7% (95%CI 5.71; 33.8) in 2014.

**Table 2:** Changes in use of antiemetics and clinical characteristics associated with hyperemesis treatment guideline update (intervention) in 2014

	**Beta- coefficient**	**95% confidence interval**
**a) Antiemetics prior to hospitalization**		
**Any antiemetic (%)**		
Trend pre-intervention, β_1_	2.52	1.17; 3.87
Level change at intervention, β_2_	-19.7	-33.8; -5.71
Trend change post-intervention, β_3_	1.98	-0.67; 4.63
Post-intervention trend, β_1_ + β_3_	4.50	1.99; 7.02
***p* < *0.001***		
**Metoclopramide (%)**		
Trend pre-intervention, β_1_	2.22	1.29; 3.14
Level change at intervention, β_2_	-30.2	-35.6; -24.8
Trend change post-intervention, β_3_	2.65	0.26; 5.03
Post-intervention trend, β_1_ + β_3_	4.86	2.68; 7.04
***p* < *0.001***		
**Prochlorperazine (%)**		
Trend pre-intervention, β_1_	0.01	-0.47; 0.49
Level change at intervention, β_2_	-0.86	-6.19; 4.47
Trend change post-intervention, β_3_	2.05	0.35; 3.75
Post-intervention trend, β_1_ + β_3_	2.06	0.48; 3.64
***p* = *0.022***		
**b) Antiemetics while at hospital**	**Beta- coefficient**	**95% confidence interval**
**Any antiemetic (%)**		
Trend pre-intervention, β_1_	1.28	0.64; 1.91
Level change at intervention, β_2_	-3.64	-9.64; 2.36
Trend change post-intervention, β_3_	-1.69	-2.86; -0.52
Post-intervention trend, β_1_ + β_3_	-0.42	-1.48; 0.65
***p* = *0.002***		
**Metoclopramide (%)**		
Trend pre-intervention, β_1_	-2.88	-5.05; -0.70
Level change at intervention, β_2_	-19.8	-36.7; -2.90
Trend change post-intervention, β_3_	1.07	-1.48; 3.62
Post-intervention trend, β_1_ + β_3_	-1.80	-2.97; -0.64
***p* < *0.001***		
**Prochlorperazine (%)**		
Trend pre-intervention, β_1_	-0.16	-2.12; 1.80
Level change at intervention, β_2_	40.5	18.2; 62.8
Trend change post-intervention, β_3_	2.86	0.51; 5.21
Post-intervention trend, β_1_ + β_3_	2.70	0.52; 4.88
***p* < *0.001***		
**c) Clinical characteristics and outcomes**	**Beta- coefficient**	**98.75% confidence interval**
**Mean gestational age at first admission (days)**		
Trend pre-intervention, β_1_	0.33	0.15; 0.49
Level change at intervention, β_2_	-3.8	-7.1; -0.55
Trend change post-intervention, β_3_	-0.24	-1.28; 0.80
Post-intervention trend, β_1_ + β_3_	0.09	-0.93; 1.10
***p* < *0.001***		
**Mean weight change at first admission (% of pre-pregnancy weight)**		
Trend pre-intervention, β_1_	-0.01	-0.09; 0.07
Level change at intervention, β_2_	0.90	-0.23; 2.02
Trend change post-intervention, β_3_	0.16	-0.14; 0.47
Post-intervention trend, β_1_ + β_3_	0.15	-0.15; 0.45
***p* = *0.0044***		
**Admission rate (% of births)**		
Trend pre-intervention, β_1_	-0.00	-0.03; 0.03
Level change at intervention, β_2_	0.11	-0.27; 0.49
Trend change post-intervention, β_3_	0.02	-0.10; 0.13
Post-intervention trend, β_1_ + β_3_	0.02	-0.10;0.13
*p* = *0.474*		
**Rate of termination of pregnancy (% of admissions)**		
Trend pre-intervention, β_1_	-0.05	-0.31; 0.21
Level change at intervention, β_2_	4.81	0.86; 8.74
Trend change post-intervention, β_3_	-0.75	-1.76; 0.25
Post-intervention trend, β_1_ + β_3_	-0.81	-1. 77; -0.15
*p* = *0.019*		

One thousand sixty-four women admitted for treatment of HG at Haukeland University Hospital 2002–2019 presented as trend 2002–2013, level change associated with updated HG-guidelines in 2014, and trend change post-intervention. Interrupted time series-analysis with Newey-West standard errors adjusting for first order autocorrelation

During hospitalization, use of metoclopramide decreased by 19.8% (95%CI 2.90; 36.7) and prochlorperazine increased with 40.5% (95%CI 18.2; 62.8) in 2014 with no impact on the proportion using any antiemetic (Fig. 5, Table 2b). Similar results were found regarding discharge prescriptions (Fig. 2c).

**Fig. 6:**
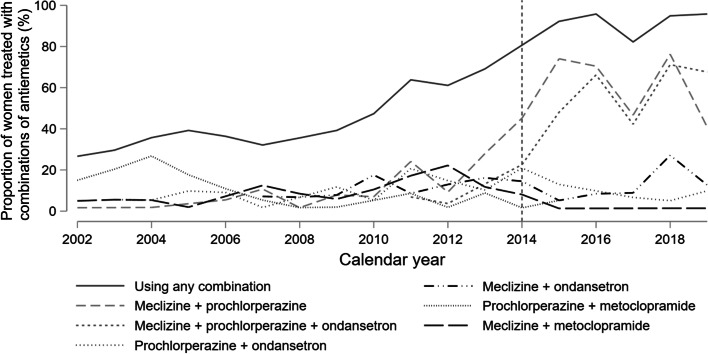
Use of combinations of antiemetic medications while hospitalized for hyperemesis gravidarum before and after 2014. Yearly proportion (%) using the six most frequently used antiemetic combinations, adding up to 76% of all combinations. Combinations defined as use of more than one antiemetic on the same day during hospital stay. Data from Haukeland University Hospital 2002–2019, *n* = 1,064

The proportion of women receiving combinations of antiemetics while hospitalized increased with 4.6% (95%CI 3.8; 5.4) yearly throughout the study period (Fig. 3). Until 2013, no apparent pattern in choice of combinations was observed (Fig. 6). A more consistent use of meclizine and prochlorperazine, and meclizine, prochlorperazine, and ondansetron was observed from 2014, and combinations including meclizine and/or prochlorperazine and/or ondansetron constituted 82% of all combinations of antiemetics used from 2014 onwards (Fig. 6).

**Fig. 7:**
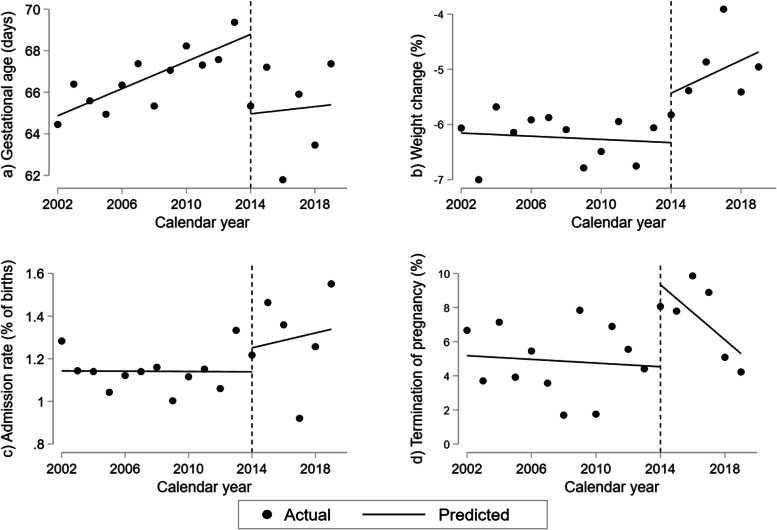
Changes in clinical characteristics and outcomes in women hospitalized for hyperemesis gravidarum 2002–2019. Interrupted time-series analyses of (**a**) average gestational age at first admission, (**b**) average weight change at first admission in percent of pre-pregnancy weight, (**c**) admission rate, and (**d**) rate of termination of pregnancy in 1,064 women hospitalized for hyperemesis gravidarum in timely association with treatment guideline update in 2014. Predicted line visualizes linear trend pre-intervention in 2014 with slope corresponding to yearly change (β_1_); vertical line illustrating level change in 2014 at the time of treatment guideline update (β_2_); predicted line after 2014 representing post-intervention trend (β_1_ + β_3_) applying Newey-West standard errors to adjust for first order autocorrelation

From the interrupted time-series analysis on clinical characteristics (Fig. 7, Table 2c), we found a drop in gestational age of 3.8 days (98.75%CI 0.55; 7.1) at first admission for HG in 2014. Additionally, a reduction in weight loss of 0.90% of pre-pregnancy weight (98.75% CI: -0.23; 2.02, *p* = 0.038), however this was not significant after Bonferroni-correction for multiple testing (*p* < 0.0125). We found no change in overall admission rate neither throughout the study period (Fig. 1) nor in the interrupted time-series analysis (Fig. 7c, Table 2c). However, the proportion of women terminating their pregnancy increased by 4.81% (98.75%CI 0.86; 8.74) in 2014. This individual element of the model remained significant despite Bonferroni-correction, but notably, the ITSA-model for terminations overall was no longer considered statistically significant (Fig. 7d, Table 2c).

## Discussion

By reviewing the patient files of women with hyperemesis gravidarum in nearly 10% of the pregnant population in Norway between 2002 and 2019, we found an overall increase in use of antiemetics both prior to, during, and after hospitalization. Following the EMA warning in 2013, restricting use of metoclopramide, we found a dramatic, but temporary, drop in the proportion of HG-patients provided antiemetics prehospitally, most notably for metoclopramide. During hospitalization, metoclopramide was persistently replaced by prochlorperazine. In timely association to the restrictions in use of metoclopramide, we observed a shift of hospitalization earlier in pregnancy, and a worrying indication of an increased proportion of HG-patients terminating their pregnancy.

### Use of antiemetic medication 2002–2019

Despite an increasing trend in use of antiemetics prior to hospitalization, 4 out of 10 women hospitalized with HG in 2019 had not used antiemetics before the condition required in-patient treatment. Among pregnant women in the UK 1998–2014, higher rates of antiemetic use were registered in women exclusively treated in primary care compared to patients hospitalized for HG, implying that pre-hospital antiemetic treatment prevents hospitalization [24]. HG treatment guidelines from the US and Canada advocate early treatment initiation to reduce need for hospital inpatient treatment, health care visits, and to alleviate the disease burden for the affected women [14, 15]. In line with European HG treatment guidelines [9, 10], we found that dopamine antagonists and antihistamines constituted nearly all pre-hospital antiemetic treatment. Metoclopramide was used by 60% of those receiving pre-hospital treatment despite not being the dopamine antagonist of choice [9, 10].

The steady increase in the use of ondansetron pre-, per-, and post-hospitalization complies with the accumulating body of evidence on safety of ondansetron in pregnancy [16, 17]. The systematic inpatient use of combinations of antiemetics including meclizine, prochlorperazine, and/or ondansetron increased in line with the Norwegian treatment guideline recommendations updated in 2014 [10]. Combinations of antiemetics with different pharmacological mechanisms of action can have synergistic effects, and are recommended also in UK guidelines for women who do not respond to monotherapy [9].

### Impact of the EMA warning on use of metoclopramide

Our findings illustrate that after the general metoclopramide recommendations from EMA and the Norwegian Health authorities with corresponding changes in the Norwegian hyperemesis guidelines, the provision of antiemetics for women suffering from HG was altered. Although in-patient use of metoclopramide was already declining, this dropped further in 2014; a trend that persisted throughout the study period. In contrast, we found a substantial, but non-persisting, drop in pre-hospital use of metoclopramide. A similar, temporary decline in metoclopramide prescription fills in pregnancy was seen from 2014 in the NorPD-study [21]. Our results suggest that the EMA warning in the HG treatment guidelines were implemented in clinical practice more extensively at the hospital compared to primary care.

Surprisingly, we found no compensatory increase in the use of other antiemetics when pre-hospital use of metoclopramide dropped, indicating that general practicioners (GPs) lacked treatment options to replace metoclopramide. This interpretation is supported by a study showing that GPs in the UK often lack confidence in prescribing recommended antiemetics in pregnancy [25], and Norwegian GPs have reported being wary of prescribing medicines for NVP due to the teratogenic potential, referring to the thalidomide tragedy [26]. The recovery in use of metoclopramide to the pre-intervention level, may suggest that GPs, in lack of alternatives, assess the benefit of metoclopramide to outweigh the potential risk of adverse effects in severe NVP/HG. Metoclopramide has thorough documentation on safety in pregnancy, and there is no suspicion of fetal malformations or other adverse pregnancy outcomes [20]. Extrapyramidal adverse reactions as adverse effects of dopamine antagonists were the reason for the EMA warning limiting metoclopramide treatment duration to five days. Paradoxically, most neurological adverse drug reactions of metoclopramide occur within the first five days of treatment [27]. Additionally, neurological adverse effects are also seen for prochlorperazine [28]. In a study on acute dystonic reactions in children, the risk was in fact higher for prochlorperazine compared to metoclopramide [29]. Thus, we suggest that women treated with any dopamine antagonist should be informed of the risk of adverse reactions, regardless of treatment duration, and advised to stop treatment if severe reactions occur.

### Changes in clinical characteristics and outcomes

Eliminating the most frequently used treatment option in primary care could be expected to increase the number of women requiring hospital treatment for HG, but no increase in hospitalization rate was observed in our cohort. This might be due to lack of power despite the relatively large cohort. However, we observed a decrease in gestational age at first admission reflecting earlier hospital referrals for HG from 2014. This change was persistent, despite recovery of the pre-hospital antiemetic provision. A possible explanation is that while use of metoclopramide pre-hospital apparently recovered after the initial drop, women were only offered five days of treatment and hospitalized if further treatment was required. In the NorPD-study, treatment duration exceeding 5 days was registered for virtually all metoclopramide prescriptions 2015–2017 [21]. However, the median treatment duration reported corresponds to the smallest package size available in Norway (20 tablets; 6.7 days), and therefore gives no indication of whether administration complied with the five-day limit. Indication of less weight loss at hospitalization in 2014, albeit not reaching statistical significance when adjusting for multiple testing, supports the interpretation that women were admitted earlier due to reduced treatment provision.

We observed a doubling in rate of termination of pregnancy in timely association with a temporary decline in pre-hospital use of antiemetics. This suggests that delayed treatment initiation might increase the risk of termination of pregnancy*.* As the Norwegian welfare system provides services such as paid sick-leave, free pregnancy care, and reimbursement for HG treatment, termination of pregnancy is less likely to be caused by practical or economic reasons compared to countries with less extensive service coverage. Reason for termination of pregnancy was not explored in this study and was likely unrelated to HG in some cases. However, the Norwegian Registry of Pregnancy Termination gives no indication of an increase in the overall number of terminations per 1000 women following the EMA warning [30], and our findings can thus not be explained by a general increase in the rate of terminations. Termination of pregnancy was associated with suicidal ideation and reduced perception of care in patients with HG in a UK study [5]. In an internet-based survey (predominantly including women from the UK and the US), 15% reported termination of pregnancy due to HG. Not being provided with antiemetics, and the providers not being caring, raised the odds of termination [31].

### Strengths and limitations

This is the largest cohort study to date in which changes in antiemetic treatment and clinical characteristics has been described from before, throughout and after hospitalization for HG. This is a well-defined hospital cohort including women with severe HG with no bias introduced by selection. A strength of the study is the level of detail of treatment information compared to registry studies where over the counter and in-patient treatment is unavailable [21]. In addition, consecutive documentation of information in routine hospital charts reduce risk of recall bias. Even though it is a single center study, the Bergen Hospital Trust catchment area covers nearly 10% of the birthing population in Norway and the findings are in line with a nation-wide antiemetic prescription registry study [21]. In addition, the study investigates consequences of a policy change concerning the whole of Europe [18] and the impact of the EMA warning on patients with NVP/HG has not been published to date. It is likely that these restrictions in antiemetic use and clinical implications identified in a Norwegian cohort might similarly affect HG patients in other European countries.

Interrupted time-series analyses are considered suitable for investigating changes in aggregated data in a population following a point of intervention [23], and has been used to investigate the effect of policy changes on metoclopramide prescriptions in Korea and the US [32, 33]. However, the ITSA-model assumes a change at the time of intervention and estimates the effects based on this assumption. Causal inference of an association between the EMA warning and associated changes in antiemetic medication provision and the clinical outcomes is limited and the results should be interpreted with caution. Adjusting the threshold of statistical significance according to the Bonferroni-correction for four outcomes reduces the risk of false positive findings but reduces power. Another limitation is that focus on treatment of HG from local research initiatives and participation in guideline development (JT) might disseminate at the department as well as the hospital catchment area, thereby influencing treatment and adherence to guidelines compared to hospitals where HG has no particular focus.

### Conclusion and future research

During 2002–2019, the overall use of antiemetics for hyperemesis gravidarum increased, while from 2014 metoclopramide in hospital was replaced by prochlorperazine. Simultaneously, a substantial, but temporary, drop in the proportion of HG-patients provided antiemetics prehospitally was observed, most notably for metoclopramide. These changes occurred in timely association with the European policy change and illustrates that the warning on metoclopramide for licensed indications led to changes in the antiemetic treatment provided for patients with HG. The concomitant tendency of hospitalization earlier in pregnancy and, in particular, the indication of an increased proportion terminating their pregnancy, suggests that these changes had negative clinical implications for women with HG. It is prudent to assess if, and to which extent, provision of timely and appropriate treatment initiation may prevent symptom progression and hospitalization, or ultimately termination of pregnancy.

## Data Availability

The datasets generated and analyzed during the current study are not publicly available as ethnical approval for dissemination has not been sought but are available from the corresponding author on reasonable request.

## Abbreviations

BMI: Body Mass Index
CI: Confidence Interval
GPs: General Practitioners
HG: Hyperemesis Gravidarum
ICD: International Classification of Disease
IQR: Interquartile Range
ITSA: Interrupted Time-series Analysis
MBRN: The Medical Birth Registry of Norway
NorPD: The Norwegian Prescription Database
NVP: Nausea and Vomiting During Pregnancy
OLS: Ordinary Least Squares
SD: Standard Deviation
